# Ice Water in Typhoid Fever

**Published:** 1884-12

**Authors:** 


**Ice Water in Typhoid Fever.**—In a clinical lecture recently delivered at the Pennsylvania Hospital, Professor Da Costa brought before the class a case of typhoid fever in which the antipyretic effects of ice-water application to the chest and abdomen had been shown to a marked degree.

The temperature in the case had been persistently high, reaching 104.4, and at one time 104.8, and rarely falling below 103, even in the morning remission. Large doses of quinine were administered, but without their usual effect. The temperature still remained high. The propriety of immersing the patient in a cold bath was then discussed, but abandoned owing to his extreme weakness, and the distance from his bed to the bath-room.

Professor Da Costa then ordered his chest and abdomen to be kept covered with cloths wrung out in ice water. This was done with the most gratifying results. The temperature fell from 104 to 102 and then to 101½. The other symptoms also began to abate.

**For Stutterers.**—A gentleman who stammered from childhood almost up to manhood gives a very simple remedy for the misfortune. Go into a room where you will be quiet and alone, get some book that will interest but not excite you, and sit down and read two hours aloud to yourself, keeping your teeth together. Do the same thing every two or three days, or once a week if very tiresome, always taking care to read slowly and distinctly, moving the lips, but not the teeth. Then, when conversing with others, try to speak as slowly and distinctly as possible, and make up your mind that you will not stammer. Well, I tried this remedy, not having much faith in it, I must confess, but willing to do most anything to cure myself of such an annoying difficulty. I read for two hours aloud with my teeth together. The first result was to make my tongue and jaws ache, that is while I was reading, and the next to make me feel as if some thing had loosened my talking apparatus, for I could speak with less difficulty immediately, The change was so great that every one who knew me remarked it. I repeated the remedy every five or six days for a month, and then at longer intervals until cured."—*Physicians and Surgeons Investigator.*

